# Anxiety and depression among epilepsy patients in low-risk areas for COVID-19 in the northern part of Guizhou Province, China, during the COVID-19 pandemic

**DOI:** 10.1186/s42494-022-00092-2

**Published:** 2022-05-10

**Authors:** Shen Wang, Juan Yang, Nian Wei, Wenbo Lv, Zhigang Jiang, Hao Huang, Jun Zhang, Ping Xu, Chang Yin Yu, Zucai Xu

**Affiliations:** 1 Department of Neurology, Affiliated Hospital of Zunyi Medical University, Zunyi, 563000 China; 2 Zunyi Medical University, Zunyi, 563000 China

**Keywords:** COVID-19, Epilepsy, Anxiety, Depression, Knowledge of epidemic prevention and control

## Abstract

**Background:**

This study was aimed to investigate whether patients with epilepsy (PWE) have higher depression and anxiety levels than the normal population in low-risk areas for coronavirus disease 2019 (COVID-19) in the northern part of Guizhou Province, China, during the COVID-19 epidemic, to evaluate their knowledge on COVID-19, and to analyze related factors for the psychological distress of PWE at this special time.

**Methods:**

The survey was conducted online from February 28, 2020 to March 7, 2020 via a questionnaire. PWE from the outpatient clinic of epilepsy of the Affiliated Hospital of Zunyi Medical University, and healthy people matched for age and sex, participated in this study. Mental health was assessed via a generalized anxiety self-rating scale (GAD-7) and the self-rating depression scale (PHQ-9). The knowledge of COVID-19 in both groups was investigated.

**Results:**

There were no significant differences in the general demographics between the PWE and healthy control groups. The scores of PHQ-9 (*P* < 0.01) and GAD-7 (*P* < 0.001) were higher in the PWE group than in the healthy group. There was a significant difference in the proportions of respondents with different severities of depression and anxiety, between the two groups, which revealed significantly higher degree of depression and anxiety in PWE than in healthy people (*P* = 0, *P* = 0). Overwhelming awareness and stressful concerns for the pandemic and female patients with epilepsy were key factors that affect the level of anxiety and depression in PWE. Further, the PWE had less accurate knowledge of COVID-19 than healthy people (*P* < 0.001). There was no statistically significant difference between the two groups in the knowledge of virus transmission route, incubation period, susceptible population, transmission speed, clinical characteristics, and isolation measures on COVID-19 (*P* > 0.05). PWE knew less about some of the prevention and control measures of COVID-19 than healthy people.

**Conclusions:**

During the COVID-19 epidemic, excessive attention to the epidemic and the female sex are factors associated with anxiety and depression in PWE, even in low-risk areas.

## Background

Epilepsy is a common disorder of the nervous system, affecting approximately 70 million people worldwide [1]. In 2017, the International Anti-Epilepsy Alliance proposed a new diagnosis and classification system for epilepsy, which clarifies the causes of epilepsy, the types of seizure and epilepsy, and furthermore, epilepsy comorbidities [2]. In recent years, in addition to the damage caused by seizures, patients also tend to develop many neurological, somatic and psychiatric comorbidities, including migraine, movement disorders, heart disease, asthma, depressive disorder, anxiety disorder, etc. These commodities of epilepsy have attracted increasing attention [3]. Compared with epileptic seizures, psychological problems such as anxiety and depression have a greater impact on the quality of life of patients with epilepsy (PWE) [4]. A study has shown the incidence of depressive disorder in 40% of PWE [5]. Comorbid mental disorders in PWE are related to poor quality of life, severity of seizures, adverse reactions to antiepileptic drugs, treatment resistance and adverse outcomes after epilepsy surgery [6–8]. However, the enormous impact of psychiatric complications in PWE is still often overlooked. The majority of patients need to take anti-seizure medicine (ASM) for a long time. Sudden discontinuation of SAM often causes a disease relapse in PWE [9]. Depression and anxiety disorders can significantly decrease the quality of life of PWE and increase the suicide rate [10].

The occurrence of public health incidents, such as the Ebola virus that is still circulating in Africa, represents a significant threat to public health and increases the risk of emotional problems. Patients recovering from Ebola have higher scores of anxiety and depression than the normal population [11]. Also noteworthy is that PWE are more susceptible to major public health incidents than the normal population. For instance, during the severe acute respiratory syndrome outbreak in 2003, PWE were reluctant to go to hospitals to buy ASMs due to the safety concerns, which led to an increase in the frequency and severity of seizures [12], further leading to psychological problems such as anxiety and depression in the patients [13]. COVID-19, a serious and potentially fatal disease that outbroke in the winter of 2019, has been spreading rapidly worldwide [14, 15]. This disease with quick and wide spread and high mortality has greatly affected human lives and induced a global prevalence of anxiety and depression. According to the official report of the World Health Organization, as of 9 June 2020, more than 7 million people worldwide have been diagnosed with COVID-19 [16]. Studies have shown that COVID-19 can cause a variety of nervous system diseases, including epilepsy, headache, disturbance of consciousness, acute cerebrovascular diseases, etc. [17]. A survey has suggested that during the COVID-19 pandemic, up to 25% and 45% of the total population have symptoms of depression and anxiety, respectively [18, 19]. The novel coronavirus causes loneliness, boredom, anger and anxiety in people and poses a serious threat to people’s physical and mental health [20]. COVID-19 has caused serious psychological problems to many people in society, such as doctors, nurses, patients and their families, who develop depression, anxiety, somatization, sleep disorders, etc. [21, 22].

Although there has been no COVID-19 outbreak in the economically underdeveloped northern part of Guizhou Province in China, sporadic cases still occurred, and local administrative departments have strictly implemented prevention and control measures. Previous studies have confirmed that depression and anxiety are more commonly seen in PWE than in the general population [23, 24]. Meanwhile, intermittent psychosis in PWE is also more common than that in healthy controls [25]. Furthermore, the prevalence of psychosis in patients with temporal lobe epilepsy (TLE) is higher than that in healthy controls [26]. Studies on factors that contribute to depression and anxiety in PWE have shown that the female sex [27], unmarried marital status, course of disease [28], seizure frequency [29], number of seizures in the past 6 months, and focal epileptic seizures [30] are risk factors for depression in PWE. In this study, we set out to assess whether the levels of depression and anxiety in PWE are higher than those in the general population during the COVID-19 outbreak, analyze the major risk factors for anxiety and depression in PWE in low-risk areas for COVID-19, and survey PWE on their knowledge toward COVID-19. The aim of this study was to help the government and the society take practical problem-solving measures for PWE and relieve their emotional stress.

## Materials and methods

### Participants

This study was a single-center, cross-sectional study. The questionnaire survey was conducted online through Wechat from February 28 to March 7, 2020. The inclusion criteria of the epilepsy group were: (1) having a history of epilepsy for at least 1 year and being followed up by neurologists every month; (2) aged > 14 years; and (3) being treated in the outpatient clinic of epilepsy of the Affiliated Hospital of Zunyi Medical University. The exclusion criteria were: (1) having nervous system diseases such as cerebral hemorrhage or a tendency of cerebral hemorrhage, intracranial tumor, or cerebral infarction; (2) having understanding disorders, obvious aphasia, communication disorders, mental retardation and hereditary mental disorders; or (3) having severe organic diseases such as liver and kidney diseases. The inclusion criteria of the healthy group were: (1) over 14 years old; (2) having good physical condition, no chronic disease; and (3) with no communication disorder or mental retardation. The exclusion criteria of the healthy group were: (1) younger than 14 years; and (2) unable to understand the questionnaire content. This study complied with the recommendations of the Ethics Committee of Affiliated Hospital of Zunyi Medical University, and was approved by the Ethics Committee of Affiliated Hospital of Zunyi Medical University.

### Content of the questionnaire

The content of the questionnaire consisted of four parts. The first part collected demographic information, including sex, age, nationality, and educational level. The second part assessed participants’ knowledge on COVID-19 and the associated control measures. The third part contained anxiety and depression scales. The generalized anxiety disorder-7 (GAD-7) is the only proven scale for PWE anxiety [31]. It is a self-reporting instrument consisting of short questions that can be completed within < 3 min. Based on the scores, the level of anxiety can be classified as normal (score of 0–4), mild (score of 5–9), moderate (score of 10–14), or severe (score of 15–21).

The Patient Health Questionnaire 9 (PHQ-9) is a screening tool for depression and has high reliability and validity in PWE [32]. According to the scores, the level of depression can be classified as no depression (score of 0–4), mild (score of 5–9), moderate (score of 10–14), moderate-to-severe (score of 15–19), or severe (score of 20–27).

The fourth part assessed patients’ adherence to antiepileptic drugs and the seizure status in the previous month before initiation of this study.

### Statistical analyses

Statistical analysis was performed using the SPSS 26.0 software. The Chi-square test was used to compare the classification variables between groups. PHQ-9 and GAD-7 scores were skewed, so the rank-sum test was used for comparison between groups. The Mann-Whitney test was used to compare non-normally distributed continuous variables. The difference was considered statistically significant when *P* < 0.05.

## Results

A total of 410 questionnaires were collected, of which 10 were excluded because the respondents were under 14 years of age. As a result, a total of 400 valid questionnaires from 200 PWE and 200 healthy subjects were analyzed.

### Clinicodemographic characteristics of the participants

Table 1 shows the general demographic data of the two groups. There was no significant difference in age, sex, educational level or nationality between the two groups. In the PWE group, 74% of the patients had focal epilepsy, while 18% had generalized epilepsy; 63% of the patients had no seizures in nearly a month prior to the initiation of the study and 65% of the patients used an ASM.

**Table 1:** Clinical and demographic characteristics of participants

Characteristics	Epilepsy patients *n* = 200		mean ± SD	Healthy controls *n* = 200		mean ± SD	*χ*^2^	*p*
	*n*	%		*n*	%			
**Age, years**			29.73 ± 10.62			29.53 ± 12.37	3.378	0.497
14–23	67	33.50		70	35.00			
24–33	77	38.50		65	32.50			
34–43	28	14.00		25	12.50			
44–53	22	11.00		31	15.50			
≥ 53	6	3.00		9	4.50			
**Sex**							0.501	0.479
Male	89	44.50		82	39.5			
Female	111	55.50		118	60.5			
**Degree of education**							3.112	0.375
Primary school	17	8.50		13	6.50			
Junior middle school	120	60.00		111	55.50			
High school	60	30.00		69	34.50			
College or above	3	1.50		7	3.50			
**Ethnic group**							0.858	0.354
Han ethnicity	162	81.00		169	84.50			
Minority	38	19.00		31	15.50			
**Number of seizures in the past month**								
0	126	63						
≤ 3	52	26						
>3	22	11						
**Epilepsy type**								
Generalized	36							
Focal	148							
Unclassified	16							
**Total number of ASM**								
1	130	65						
>1	70	35						

### Comparison of depression and anxiety between the two groups

The PHQ-9 (*P* < 0.01) and GAD-7 (*P* < 0.001) scores in the PWE group were higher than those in the control group (Table 2). In addition, there was a significant difference in the proportions of respondents with different severities of depression and anxiety, between the two groups, which revealed significantly higher degree of depression in PWE than in the healthy people (*P* = 0) (Table 3).

**Table 2:** Scores of the depression and anxiety scales

***n***	GAD-7			Z	*P*	PHQ-9			Z	*P*
	Min	Max	Median (P25, P75)			Min	Max	Median (P25, P75)		
Epilepsy patients 200	0	21	2 (0,6)	−4.80	<0.05	0	24	2 (0,8)	−3.76	<0.05
Healthy controls 200	0	16	0 (0,3)			0	22	0 (0,4)		

*GAD-7* Generalized Anxiety Disorder-7, *PHQ-9* Patient Health Questionnaire 9

**Table 3:** Comparison of proportions of participants with different severity of depression and anxiety between the two groups

	Depression severity					Z	*P*	Anxiety severity				Z	*P*
	None	Mild	Moderate	Moderae and severe	Heavily severe	−3.616	0	None	Mild	Moderate	Heavily severe	−3.621	0
**Epilepsy patients**	123 (61.5%)	34 (17%)	25 (12%)	11 (5.5%)	7 (3.5%)			138 (69%)	32 (16%)	18 (9%)	12 (6%)		
**Healthy controls**	154 (77%)	28 (14%)	10 (5%)	7 (3.5%)	1 (0.5%)			167 (83.5%)	23 (11.5%)	8 (4%)	2 (1%)		

### Comparison of PHQ-9 and GAD-7 scores between subgroups of PWE

The PHQ-9 and GAD-7 scores of the females in the epilepsy group were higher than those of the males (*P* < 0.01) (Table 4). The PWE who were concerned about the pandemic had higher scores of depression and anxiety (*P* < 0.05). However, there were no significant differences in anxiety and depression scores between AWE of different ages, between AWE who considered life was disturbed and undisturbed, between AWE taking single ASM and multiple ASMs, and between AWE who had epileptic seizures in the past month and who did not (*P* > 0.05).

**Table 4:** Comparison of PHQ-9 and GAD-7 scores within the epilepsy group

	Epilepsy patients *n* = 200		PHQ-9	Z/χ^2^	*P*	GAD-7	Z/χ^2^	*P*
	*n*	%	Median (P25, P75)			Median (P25, P75)		
**Sex**				−3.263	0.001		−2.535	0.011
Male	89	44.50	1 (0, 5)			0 (0,4)		
Female	111	55.50	4 (0, 10)			3 (0,8)		
**Age, years**				4.408	0.354		6.451	0.168
14–23	67	33.50	4 (0, 11)			2 (0,9)		
24–33	77	38.50	2 (0, 7)			1 (0, 5)		
34–43	28	14.00	3 (0.25, 10)			3.5 (0, 8.5)		
44–53	22	11.00	0 (0, 6.5)			0 (0, 4.25)		
≥ 54	6	3.00	3.5 (0.75, 9.75)			3 (0, 8)		
**Do you pay attention to the COVID-19’s epidemic situation every day?**				−3.102	0.002		−2.287	0.022
Yes	182	91.00	2 (0, 8)			1 (0, 6)		
No	18	8.00	7.5 (4, 10.5)			4.5 (1.75, 9.5)		
**Is life disrupted?**				−1.21	0.226		−1.423	0.155
Yes	118	59.00	4 (0, 8)			3 (0, 5)		
No	82	41.00	1 (0, 8.25)			1 (0, 5)		
**Type of medication**				−0.098	0.922		−1.556	0.12
**A single ASM**	130	65.00	2 (0, 8)			2 (0, 5)		
**A combination of ASMs**	70	35.00	2.5 (0, 10.25)			2.5 (0, 7.25)		
**Did you have seizures in the past month?**				−0.91	0.363		−1.08	0.28
Yes	74	37.00	3 (0, 9.25)			3 (0, 7)		
No	126	63.00	2 (0, 7.25)			1 (0, 5.25)		

*GAD-7* Generalized Anxiety Disorder-7, *PHQ-9* Patient Health Questionnaire 9, *ASM* Anti-seizure medicine

### Comparison of knowledge on COVID-19 between the PWE and control groups

The PWE and control groups both had high accuracy in knowledge on the source of infection (confirmed patients and asymptomatic infection), route of transmission (such as respiratory droplets and contact transmission), incubation period, clinical manifestations (fever, fatigue, dry cough), isolation measures, treatment and prognosis of COVID-19. The PWE group had a lower rate of correct answer regarding the need for negative results of two nucleic acid tests (with sampling interval of at least 1 day) for exclusion from suspected cases, compared to the normal group. Both groups had a high rate of correct answer for the use of 75% alcohol and 84 disinfectant to effectively inactivate the virus, and had a high awareness rate of reducing the number of parties and taking personal protective measures (handwashing, wearing masks, etc.). Among the relevant measures for the prevention and control of COVID-19, staying at home to prevent infection, wearing masks when going out, washing hands frequently, seeking medical treatment, actively isolating after contact with high-risk groups, covering the mouth and nose when coughing or sneezing, and paying attention to keeping warm are measures that were recognized by participants of both groups. Further, the PWE group had a lower recognition rate regarding monitoring of body temperature, proper exercise, and disinfection of the home environment. Besides, the normal control group had a very high awareness rate of other personal protection measures, except the home environment disinfection measure (Tables 5 and 6).

**Table 5:** Comparison of knowledge on COVID-19 prevention and control measures between the two groups

Question	Always		Often		Occasionally		Never		Z	*P*
	Epilepsy patients *n* (%)	Healthy controls *n* (%)	Epilepsy patients *n* (%)	Healthy controls *n* (%)	Epilepsy patients *n* (%)	Healthy controls *n* (%)	Epilepsy patients *n* (%)	Healthy controls *n* (%)		
Stay at home after the outbreak to prevent infection	160 (80)	172 (86.00)	37 (18.5)	21 (10.50)	1 (0.5)	5 (2.50)	2 (1.00)	2 (1.00)	−1.467	0.142
Wear a mask when you go out	177 (88.5)	198 (99.00)	17 (8.5)	2 (1.00)	5 (2.5)	0 (0)	1 (0.5)	0 (0)	−4.343	<0.001
Wash hands frequently	163 (81.5)	188 (94.00)	37 (18.5)	11 (5.50)	0 (0)	1 (0.50)	0 (0)	0 (0)	−3.779	<0.001
When you have a fever, cough and other symptoms, you will see a doctor in time	158 (79)	184 (92.00)	27 (13.5)	12 (6.00)	10 (5)	4 (2.00)	5 (2.5)	0 (0)	−3.742	<0.001
Monitor body temperature	83 (41.5)	145 (72.50)	79 (39.5)	35 (17.50)	31 (15.5)	19 (9.50)	7 (3.5)	1 (0.50)	−5.963	<0.001
Open the window for ventilation to keep the air fresh	130 (65)	184 (92.00)	66 (33)	15 (7.50)	3 (1.5)	1 (0.50)	1 (0.5)	0 (0)	−6.550	<0.001
Have a reasonable rest and do not stay up late	128 (64)	156 (78.00)	53 (26.5)	29 (14.50)	11 (5.5)	13 (6.50)	8 (4)	2 (1.00)	−2.955	0.003
Proper exercise	101 (50.5)	147 (73.50)	65 (32.5)	26 (13.00)	31 (15.5)	25 (12.50)	3 (1.5)	2 (1.00)	−4.174	<0.001
Home environment disinfection	73 (36.5)	123 (61.50)	88 (44)	43 (21.50)	33 (16.5)	30 (15.00)	6 (3)	4 (2.00)	−4.113	<0.001
Reduce the environment of de-sealing and poor ventilation	129 (64.5)	177 (88.50)	37 (18.5)	20 (10.00)	12 (6.00)	3 (1.50)	22 (11)	0 (0)	−5.973	<0.001
Reduce de-sealing and poor ventilation in the environment	136 (68)	181 (90.50)	32 (16)	15 (7.50)	10 (5)	3 (1.50)	22 (11)	1 (0.50)	−5.752	<0.001
Avoid direct contact with public facilities that may be infected, such as elevator buttons, stair handrails, etc.	126 (63)	161 (80.50)	38 (19)	31 (15.50)	16 (8)	8 (4.00)	20 (10)	0 (0)	−4.360	<0.001
Take the initiative to isolate after contact with high-risk groups	150 (75)	189 (94.50)	20 (10)	9 (4.50)	18 (9)	1 (0.50)	12 (6)	1 (0.50)	−5.551	<0.001
Cover nose and mouth when coughing or spraying sneezing	151 (75.5)	188 (94.00)	37 (18.5)	12 (6.00)	8 (4)	0 (0)	4 (2)	0 (0)	−5.228	<0.001
Keep warm and avoid catching cold	157 (78.5)	190 (95.00)	39 (19.5)	10 (5.00)	2 (1)	0 (0)	2 (1)	0 (0)	−4.887	<0.001

**Table 6:** Comparison of knowledge regarding COVID-19 between the two groups

Question	Epilepsy patients *n* = 200	Healthy controls *n* = 200	*χ*^2^	*P*
	Correct *n* (%)	Correct *n* (%)		
The source of infection is confirmed patients and asymptomatic infections.	175 (87.5)	191 (95.5)	8.229	0.004
Mainly transmitted through respiratory droplets and contact	197 (98.5)	199 (99.5)	1.010	0.315
People are generally susceptible to infection.	187 (93.5)	190 (95.0)	0.415	0.519
Highly contagious and fast transmission	198 (99)	199 (99.5)	0.336	0.562
Infection can be effectively reduced by frequently washing hands and wearing masks.	199 (99.5)	198 (99.0)	0.336	0.562
Family gatherings can infect each other.	183 (91.5)	188 (94.0)	0.929	0.335
The incubation period of the disease is 1–14 days, mostly 3–7 days.	187 (93.5)	193 (96.6)	1.895	0.169
Most of the infected people are characterized by fever, fatigue and dry cough.	199 (99.5)	200 (100)	1.003	0.317
Most of the patients have a good prognosis and a few are in critical condition.	185 (92.5)	194 (97.0)	4.071	0.044
Suspected cases can only be excluded if the respiratory pathogen nucleic acid test is negative for two consecutive times (the sampling time is at least 1 day apart).	140 (70)	171 (85.5)	13.888	0
Suspected and confirmed cases should be isolated and treated in designated hospitals with effective isolation and protective conditions.	197 (98.5)	200 (100)	3.023	0.082
When you have a fever during the epidemic period, you are not allowed to take antipyretic drugs on your own.	180 (90)	184 (92.0)	0.488	0.485
75% of alcohol can effectively inactivate the virus.	167 (83.5)	190 (95.0)	13.784	0
84 disinfectant can effectively inactivate virus	166 (83)	159 (79.5)	0.804	0.370

### Anxiety and depression scores of people who cared about COVID-19 in both groups

The patients in the epilepsy group who cared about COVID-19 daily (*n* = 182) had significantly higher anxiety and depression scores than those in the normal group (*n* = 190) (*P* < 0.05, Table 7).

**Table 7:** Anxiety and depression scores for people who care about COVID-19 in the two groups

	*n*	GAD-7 Score Min Max Median (P25, P75)			Z	*P*	PHQ-9 Score Min Max Median (P25,P75)			Z	*P*
**Epilepsy patients**	182	0	21	2 (0, 6)	−4.803	<0.001	0	24	2 (0, 8)	−3.736	<0.001
**Healthy controls**	190	0	16	0(0,3)			0	22	0(0,4)		

*GAD-7* Generalized Anxiety Disorder-7, *PHQ-9* Patient Health Questionnaire 9

## Discussion

PWE are likely to suffer mental disorders. The influencing factors for emotional problems in PWE include sex [27], age, frequency of seizures and the type of anti-seizure drugs [29, 30]. In this study, we found that in the COVID-19 epidemic, PWE patients had significantly higher levels of anxiety and depression than the normal group, which was closely related to the attention of PWE to the epidemic and the lack of knowledge on COVID-19 prevention and control. The influencing factors were different from previously reported.

Given the severe morbidity and high mortality in epilepsy patients, the ongoing COVID-19 pandemic has placed an unprecedented stress on the patients, and even more serious social and economic impact than natural disasters such as earthquakes and hurricanes [33].

In this study, we described the mental health status of PWE in the northern part of Guizhou Province during the COVID-19 pandemic, in comparison with the healthy group, and analyzed the related risk factors for PWE mental state fluctuations during the COVID-19 pandemic. We found that 38.5% of PWE had depression and 31% of PWE had anxiety, a rate significantly higher than that in the healthy group. Some studies have shown that over 13% of PWE experienced severe mental stress during the COVID-19 pandemic. The daily amount of time PWE spent on getting information of COVID-19 from the media was associated with the severity of psychological distress in PWE during seizures [34]. Consistently, here we found that PWE who paid too much attention to the COVID-19 pandemic might experience psychological problems. Altogether, these studies confirm that PWE are more likely to suffer from comorbid mental disorders [35]. In addition, there is clear evidence that the increase in seizure frequency and severity could improve the prevalence of anxiety and depression in PWE [13, 36].

We found that PWE who had a daily follow-up on the COVID-19 pandemic were more prone to anxiety and depression. The input of COVID-19 epidemic information from social media, TV news, radio and other means adds to their psychological burden [37]. A meta-analysis has shown that there is no significant relationship between epilepsy and severe psychological problems in the absence of international public health emergency [38, 39]. Here, we also found that female PWE were more susceptible to comorbid anxiety and depression, which is consistent with previous reports [40, 41].

Another factor affecting the mental state of PWE during the pandemic was whether their lives are disrupted. The levels of depression and anxiety of PWE who felt their lives were disturbed were significantly higher than those of PWE who felt no disturbance. It is worth noting that the PWE had normal psychological coping and adjustment abilities. The COVID-19 outbreak occurred during the Chinese New Year Festival, which resulted in cancelation of all celebrations, shut-down of transportation, businesses and public entertainments, and blockade of family reunion. All of the restrictions would cause greater psychological distress in PWE.

In the other survey, we found a lower proportion of PWE with correct knowledge on COVID-19 than that in the healthy group, which may be due to the knowledge and education of PWE, or because that epilepsy patients paid too much attention to their diseases and were not interested in COVID-19 knowledge. The lack of awareness of COVID-19 may also cause panic, anxiety and depression in PWE. Cross-sectional studies have also confirmed that the lower the level of education, the greater the risk of depression [23]. The reason may be that PWE have a lower educational level, poor working environment, a low or a loss of working ability, and low income, which lead to the poor control of epilepsy and make them more prone to depression and other psychological problems. This pandemic has also had an impact on the health care systems in many countries, with an inevitable gap in the accessibility of treatment of chronic diseases, including epilepsy. The COVID-19 pandemic has delayed the diagnosis and treatment of some patients, which poses a threat to the mental health of PWE [42]. Epilepsy, as a kind of mental stress, is closely related to major public health events [43].

This study had some limitations. With a cross-sectional design, the study did not track the psychological status of the two groups of subjects, nor did it track whether new mental disorders developed as the epidemic progressed. In addition, the psychological assessments were based on online surveys and self-report tools. In future studies, the use of clinical interviews will be encouraged to allow for a more comprehensive assessment.

## Conclusions

During a public health outbreak, healthcare professionals should focus not only on seizure control but also on the mental health of the PWE. The PWE need more psychological guidance, and the government should provide more psychological assistance to PWE while taking preventive and control measures.

## Data Availability

All data generated or analyzed in this study are included in this manuscript.

## Abbreviations

ASM: Anti-seizure medicine
COVID-19: Coronavirus disease 2019
GAD-7: Generalized anxiety self-rating scale
PWE: Patients with epilepsy
PHQ-9: Self-rating depression scale
TLE: Temporal lobe epilepsy
